# Some Experiments with Muriate of Cocaine in the Dental Infirmary of the University of Maryland

**Published:** 1885-02

**Authors:** Chas. L. Steel

**Affiliations:** Demonstrator of Operative Dentistry.


SOME EXPERIMENTS WITH "MURIATE OF COCAINE" IN THE DENTAL INFIRMARY OF THE UNIVERSITY OF MARYLAND. Chas. L. Steel, M. D., D. D. S., Demonstrator of Operative Dentistry.—As soon as this new aæsthetic was brought to the notice of the profession, experiments were introduced with in the Dental Infirmary of the University of Maryland. At first we could only procure a 2 per cent. solution. This was tried for extracting but with very little effect. Subsequently the 4 per cent. solution was tried, but with little if any improvement. About this time, Messrs. McKesson & Robbins very kindly supplied us with a sample of their 5 per cent. oleate. It is a well known fact that most medicines in the form of oleates permeate the tissues more readily than in any other form. I therefore hoped to find marked improvement in the degree of anæsthesia produced by the use of this preparation—nor was I *entirely* disappointed. I am sure that I have produced complete anæsthesia of the *gum* with the oleate. In one instance a very firmly retained temporary tooth was removed, *apparently*, without *any* pain—in extracting roots, with little or no bony attachment, the anæsthesia was very decided. When we come, however, to use it in the extraction of teeth more firmly implanted, I consider it as almost valueless. The pain from the extraction of a tooth is deep-seated for an agent, so notably superficial in its action as is cocaine, to be of material benefit. Possibly we may sometimes discover some method of application by which it will be more effective. I applied the oleate with a camel's hair brush, keeping the gum as dry as possible, and painting with the oleate seme six or eight times in the course of ten minutes. In using the cocaine for obtunding sensitive dentine, the experiments were much more satisfactory. A student came to me one day complaining that he had, with much trouble, received the decay from two shallon, saucer-shaped canties, but that the patient was so nervous and the teeth apparently so sensitive, that he could not properly prepare the canties for the retention of the fillings. I directed him to put on the rubber dam, and as soon as this was done, I dropped a little of the 4 per cent. solution in the canties and in about five minutes, the dental eugine was used, the canties nicely prepared and *no pain experienced by the patient.* In numerous other cases, it was used for sensitive dentine, with varying successes, but always with decided advantage. We are not always safe to rely upon the statements of patients and I am therefore glad to be able to record the testimony of a student, in whose tooth it was used, as they (students) can usually speak more intelligently about these matters, than an ordinary patient can. Mr. C. had a cavity in the anterior approximal surface of a lower molar. It was so sensitive that he had never been able to stand the pain of attempted excavation. By repeated applications of the cocaine the cavity was finally prepared most satisfactorily and although some pain was felt yet it was greatly alleviated.

Unquestionably, in my opinion, cocaine is the best ob-tunder of sensitive dentine, which we now have, but the question still remains to be proven ; will it produce any tasting and injurious effect on a nerve which might be preserved if this drug were not used ?

In another existance the cocaine was used most successfully by a student in extirpating a large fungous growth from a tooth.

In my private practice I have used it quite extensively. In one case, I succeeded in extirpating a live nerve, *absolutely without pain.* In this case, I used the pure alkaloid, applied on a piece of moistened cotton.

The drug is as yet too young to have its place Dentistry, distinctly defined, but that it will have a prominent position in our medicine case, I doubt not.

